# The functional diversity of the POUV-class proteins across vertebrates

**DOI:** 10.1098/rsob.220065

**Published:** 2022-06-29

**Authors:** Evgeny I. Bakhmet, Alexey N. Tomilin

**Affiliations:** Laboratory of the Molecular Biology of Stem Cells, Institute of Cytology, Russian Academy of Sciences, St Petersburg, Russia

**Keywords:** Pou5f3, Pou5f1, Oct4, pluripotency, ESCs, embryogenesis

## Abstract

POUV is a relatively newly emerged class of POU transcription factors present in jawed vertebrates (Gnathostomata). The function of POUV-class proteins is inextricably linked to zygotic genome activation (ZGA). A large body of evidence now extends the role of these proteins to subsequent developmental stages. While some functions resemble those of other POU-class proteins and are related to neuroectoderm development, others have emerged *de novo*. The most notable of the latter functions is pluripotency control by Oct4 in mammals. In this review, we focus on these *de novo* functions in the best-studied species harbouring POUV proteins—zebrafish, *Xenopus* (anamniotes) and mammals (amniotes). Despite the broad diversity of their biological functions in vertebrates, POUV proteins exert a common feature related to their role in safeguarding the undifferentiated state of cells. Here we summarize numerous pieces of evidence for these specific functions of the POUV-class proteins and recap available loss-of-function data.

## Graphical abstract

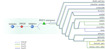

## 1. Introduction

The POUV class consists of a group of proteins that harbour the POU domain and are expressed mainly during early embryogenesis [1,2]. The most famous member of this class is Oct4, a key factor for induction and maintenance of pluripotency [3–5]. Considering the impact of this protein on these processes, it can be assumed that Oct4 orthologues exhibit strong conservation among multicellular organisms. Surprisingly, though, members of the POUV class were found only in vertebrates and, remarkably, only in jawed vertebrates (Gnathostomata), from cartilaginous fishes to human, and not in lampreys, for example [6,7]. In our previous review, we focused on the structural features of Oct4 and the POUV class and the role of these proteins in pluripotency induction and zygotic genome activation (ZGA) [8]. We concluded that although POUV-class proteins make a major contribution to ZGA in anamniotes (zebrafish, *Xenopus*), they are overtaken by proteins such as Nfya and Dux in Placentalia development. Nevertheless, POUV proteins from all studied vertebrates are characterized by numerous functions in embryogenesis following ZGA. Some of these functions, which are performed by Pou5f3 in zebrafish and *Xenopus*, are indicative of the origin of POUV from the POUIII class and are related to neuroectoderm development—midbrain–hindbrain boundary establishment, embryo integrity and neuro-progenitors maintenance [9–14]. Mammals possess Pou5f1 (Oct4), a protein known primarily as a key regulator of pluripotency—the undifferentiated state that endows cells the ability to become endo-, ecto- and mesoderm [3,15–17]. Interestingly, both Pou5f3 and Pou5f1 have a common function in posterior (trunk and tail) body extension [9,11,18,19], a feature that may be indicative of the role of POUV in maintenance of the undifferentiated state. This state is needed for regulative development of vertebrates and for prevention of premature differentiation to one or another trajectory. However, in some cases, Oct4 at least does not prevent differentiation, suggesting that the function of this transcription factor is context dependent. In this review, by summarizing available loss-of-function data, we focus on the post-ZGA functions of the POUV class during early embryogenesis of jawed vertebrates, denoting both conserved and newly emerged functions.

## 2. POUV-class origin

**Figure 1.**
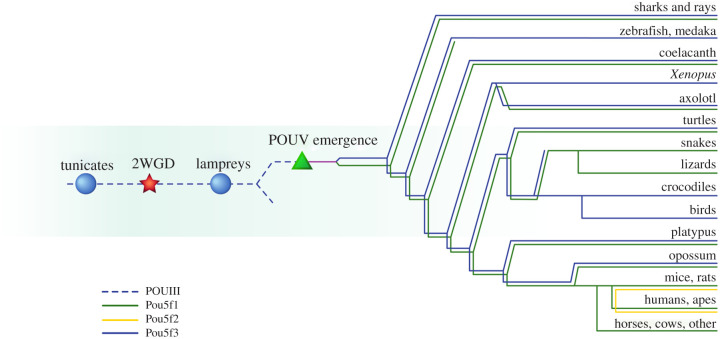
Origin and diversification of POUV-class proteins in evolution. The figure is modified from [22] and [7]. Dashed line represents a POUIII-class ancestor, which existed before the emergence of the POUV class. 2WGD represents two subsequent whole-genome duplications.

The POU domain consists of the combination of a POU-specific subdomain (POUs), a linker and a homeodomain (POUh) [1]. POU-domain proteins emerged after the divergence of choanoflagellate and animals, but before the divergence of sponges and eumetazoans. Thus, POU-domain proteins are not present in plants and fungi, and like other metazoan-specific proteins such as Six and Pax, they appear to have contributed to animal multicellularity [20,21]. The POUV-class proteins evolved approximately 450 million years ago in some jawed ancestor (Gnathostomata) and across different taxa, represented by two orthologues—Pou5f1 (Oct4) and Pou5f3 (previously known as pou2) (figure 1) [6,22,23]. The evolution of the POUV class was reviewed in detail elsewhere [7,24,25]. All studied jawed vertebrates bear at least one of these orthologues and demonstrate an early lethal phenotype upon Pou5f1 or Pou5f3 factor knockout [17,26,27]. Considering that POUV-class members are involved in important processes during early embryonic development, it is surprising that they appeared during evolution only relatively recently [28]. The appearance of POUV class in vertebrates could be related to global rearrangements in the genome, resulting in the emergence of principally new organisms. Whole-genome duplications (WGDs), which occurred before the origin of vertebrates, is an example of such a rearrangement [29–32]. Two subsequent WGDs took place between tunicates and lampreys [33,34] and perhaps account for the switching from a ‘mosaic’ type of development to a ‘regulative’ development. The former type of development is typical for most invertebrates while the latter applies to all vertebrates. However, the absence of any POUVs in the lamprey's genome [35] does not support the role of WGDs in the origin of the POUV class. Thus, unless lampreys had *POUV* genes and then lost them, this class likely appeared by simple duplication of some *POUIII* gene (figure 1) [36]. This is confusing in light of the relatively similar early development of lamprey and zebrafish [37,38] as well as the indispensability of POUV-class proteins for embryogenesis of all vertebrates except lampreys (discussed below). Nevertheless, other POU-domain classes are widely distributed across multicellular organisms (classes I, III, IV and VI) probably first appearing at the dawn of Metazoa development and, now present in species ranging from sponges to human [20,21,36]. It is unlikely that these proteins perform POUV functions because they are not related to early embryogenesis [39]. Members of the POUIII class, which is probably the ancestor of POUV, usually serve as regulators of neuroectoderm development [40–45]. Interestingly, the emergence of Nanog is also associated with jawed vertebrates, as Nanog has been found as early as in Osteichthyes [46]. Of note, the SoxB class, which in cooperation with POUV proteins regulates early development and maintenance of pluripotency, has existed much longer than the POUV class and was already present in sponges [28,47,48]. Also, the partnership between Sox and POU proteins could have existed longer than the Sox-POUV partnership, even as early as in invertebrates. For example, it was proposed that the cooperation between the HMG-containing *Dichaete* and the POU protein *vvl* occurs during *Drosophila* neurogenesis [49–51]. On the other hand, *in vivo* data (ChIP-seq) show that DNA-dependent formation of the Sox–Oct dimer is more typical for POUV-class proteins, while other POU factors prefer binding to DNA as homodimers [52–55].

Although it is generally accepted that participation of POU-domain proteins in pluripotency is a privilege of Gnathostomata, several reports have attempted to address roles of these proteins in stem cell function in invertebrates [56–59]. It was shown that the cnidarian POU-containing protein Pln, which is likely to be a POUVI-class member, is expressed in the embryo and adult stem cells (i-cells) [59]. Surprisingly, these cells could be positively stained with anti-human Oct4 antibodies. However, considering that Pln is not even an orthologue of Oct4 but rather a paralogue from another POU class, this observation casts some doubts. Oocytes, embryos, primordial germ cells and some branchial sac cells in the tunicate *Botryllus schlosseri* are stained positively by anti-Oct4 antibodies [56], which is also inconsistent with the absence of Oct4 orthologues in tunicates. The study of planarian stem cells has revealed evolutionary conservation of a gene network governing pluripotency between these organisms and mammals, including genes affecting both Oct4 and Oct4 target expression [58]. Considering the ambiguity of this data, additional research on the potential stem cell function of POU-domain proteins is needed. To date, involvement of other proteins such as Piwi and Vasa in invertebrate stem cell function represents a more likely scenario [60].

## 3. Biological functions of POUV proteins in anamniotes

### 3.1. Zebrafish

**Figure 2.**
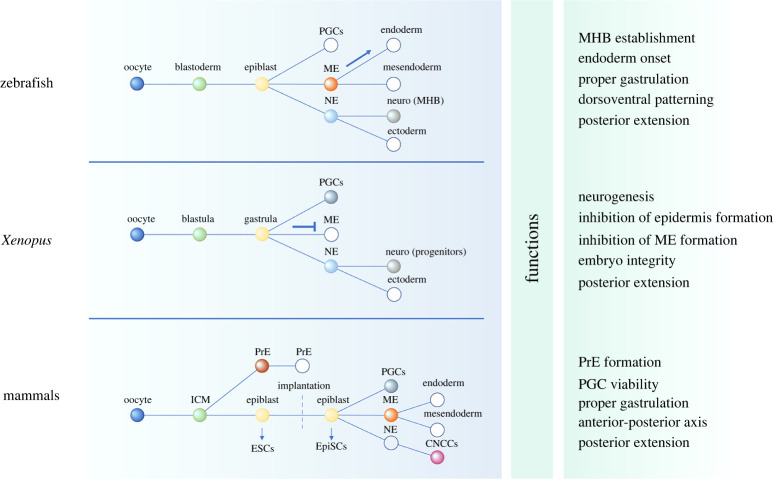
POUV protein expression and functions during embryogenesis in zebrafish, *Xenopus* and mammals; non-coloured points denote the absence of POUV expression at this stage, while colour-filled circles denote POUV expression at this stage; different colours reflect POUV expression at comparable stages of development across taxa; arrow denotes involvement of POUV in the specification of certain tissue, whereas blunt arrow denotes inhibition of tissue specification by POUV. ICM, inner cell mass; PGCs, primordial germ cells; ME, mesendoderm; NE, neuroectoderm; MHB, midbrain–hindbrain boundary; PrE, primitive endoderm; ESCs, embryonic stem cells; EpiSCs, epiblast stem cells; CNCCs, cranial neural crest cells.

These animals are the most studied early Gnathostomata across vertebrates. Their POUV member Pou5f3 is known to be a regulator of neuroectoderm and endoderm development, as well as an organizer of dorsoventral patterning and gastrulation, acting via regulation of cell motility. Maternally expressed Pou5f3 is present in the zygote, whereas embryonic Pou5f3 begins to be expressed in the blastoderm and becomes restricted to the epiblast. Subsequently, Pou5f3 is expressed in the midbrain and hindbrain at late gastrulation and early somitogenesis (figure 2) [9,61]. While there is no evidence of Pou5f3 expression in zebrafish primordial germ cells (PGCs) [62], medaka fish PGCs were shown to harbour expression of this protein [63,64].

Early Pou5f3 research in zebrafish showed that this protein is necessary for establishment and maintenance of the midbrain–hindbrain boundary (MHB) organizer [9,10,18]. MHB is responsible for proper neuroectoderm patterning and differentiation, and it is dependent on Fgf8, Wnt1 and Pax2.1 to exert its function. These markers demonstrated strong decline in expression upon *Pou5f3* knockdown and consequently, several morphological defects in neurogenesis were observed—no MHB, a smaller midbrain, and fewer neurons in the spinal cord. Defects in trunk and tail development during *Pou5f3* knockdown were also noted—abnormal somite morphology and variable tail length [9,18]. Furthermore, using embryos with maternally and zygotically knocked-out *Pou5f3* (MZspg), MHB defects became more pronounced; however, an earlier phenotype characterized by gastrulation delay and endoderm loss was discovered [26,65]. It was shown that Pou5f3 maintains Nodal-dependent *Sox32* (cas) expression; together, Pou5f3 and Sox32 activate *Sox17* transcription, a requirement for endoderm development. Pou5f3 and Sox32 bind cis-regulatory modules B and C of *Sox17* gene, respectively, and act synergistically [66]. Accordingly, MZspg mutants had reduced Sox32 (cas) and Sox17 levels and failed to develop endoderm tissue. Gastrulation delay is also a distinctive feature of MZspg. By the time wild-type embryos reach 30% epiboly, the mutants reach only the dome stage [26]. This abnormality is related to defects in cytoskeleton, cell adhesion, and cell behaviour in MZspg [67]. It was shown that both upward and downward intercalations during gastrulation are significantly affected in *Pou5f3*-null embryos [68]. These abnormalities in cell motility are related to disturbances in E-cadherin endosomal trafficking; while this molecule is used during epiboly in wild-type embryos, in MZspg it accumulates on the plasma membrane and interferes with cell mobility. E-cadherin endocytosis is controlled by Pou5f3-dependent *EGF* expression and thus, ectopic EGF mRNA could rescue E-cadherin distribution in MZspg [69]. *Pou5f3* deficiency has also shown an influence on cell viability during gastrulation and is related to *mych* activity [70]. Using ChIP-seq analysis, Kotkamp *et al.* showed that transcription of both *mych* and *mycl1b* is directly regulated by Pou5f3. MZspg mutants showed increased apoptosis during gastrulation, and this phenotype was partially rescued by ectopic *mych* expression, whereas combined *mych* and *p53* overexpression completely attenuated apoptosis in MZspg [70]. The authors also showed a specific role for Pou5f3 in activating *Klf2a*, *Klf2b* and *Klf17* during the establishment of the extraembryonic envelope layer (EVL) and ectoderm. In the case of the ventral ectodermal domain, Pou5f3 acts together with BMP to mediate *Klf2a* and *Klf2b* expression [71]. Finally, there is the well-known function of Pou5f3 in the dorsoventral patterning of the zebrafish embryo, again achieved through collaboration with BMP signalling [62,72,73]. MZspg mutants are characterized by dorsalization and ventral expression of BMP antagonists Gsc, Chd and Nog1. Pou5f3 promotes ventralization through activation of Bmp2b, Bmp4, Bmp7, Vox, Vent and other factors. Ventralization is at least partly achieved via the Alk8-TGFbeta receptor, as receptor overexpression was shown to rescue *Bmp2b* and *Bmp4* activation. It was also demonstrated that Pou5f3 directly regulates Vox transcription through a specific regulatory element [73].

Considering that lampreys resemble zebrafish in early development but have no POUV analogues, it is surprising that this new class of proteins in zebrafish has acquired so many functions. It seems that this may be due, at least partially, to participation of the newly emerged POUV class in zygotic genome activation [52,74,75], and some of the discussed defects in MZspg mutants could be related to failure to initiate the expression of one or several key developmental regulators. Therefore, the major mutant phenotype is characterized by gastrulation delay and consequent absence of endoderm—and may be caused by genome activity shutdown. At the same time, the listed defects in neurogenesis may indicate the POUV origin—the neuroectoderm regulators of the POUIII class.

### 3.2. Xenopus

Amphibians, like fish, are anamniotes and have similar early development. However, the function of POUV proteins in these animals is different from and sometimes even opposite to that in zebrafish. Of note, *Xenopus*, unlike fish and mammals, does not express Nanog, and Ventx is likely to serve the role of Nanog in this species [46]. The POUV class in *Xenopus* is represented by three Pou5f3 homologues—Pou5f3.1 (Oct91/Xlpou91), Pou5f3.2 (Oct25/Xlpou25) and Pou5f3.3 (Oct60/Xlpou60). In addition to their role in genome activation [76], these Pou5f3 proteins perform several functions in neurogenesis and cell integrity. Their expression pattern is different throughout early embryogenesis. *Pou5f3.3* is maternally expressed and is downregulated in blastula and undetected early during gastrulation [11]. *Pou5f3.1* and *Pou5f3.2* are expressed after ZGA in animal and marginal blastula zones and are then expressed during gastrulation (not in involuting cells) and in the developing neural tissue (figure 2) [11]. Pou5f3.1 (Oct91, Xlpou91), which completely rescues Oct4-null mouse embryonic stem cells (ESCs) [11], is not maternally expressed and functions after genome activation. However, like mouse Oct4, this protein is found in *Xenopus* PGCs [77].

Like zebrafish, in *Xenopus*, POUV proteins play a notable role in neurogenesis. Pou5f3.1 and Pou5f3.2 downregulation leads to a decline of neural markers Fgf8, En2 and Krox20, as well as upregulation of organizer (Cer, Gsc, Chordin) and endoderm (Sox17, Mixer, Endodermin) markers [11]. This observation is unexpected due to the crucial role of POUV in endoderm formation of the evolutionarily older zebrafish and in primitive endoderm formation of the evolutionarily more recent mammals (discussed below). On the other hand, the authors speculate that their results may indicate a conserved role of POUV-class proteins in prevention of premature commitment, which is supported by the ability of these proteins to rescue the self-renewal of mouse ESCs [11]. The participation of Pou5f3.1 and Pou5f3.2 in neurogenesis occurs in part through activation of *Chch* and *Sip1*, as overexpression of these two proteins rescues Pou5f3.1-knockdown embryos [12]. Interestingly, Pou5f3.1 and Pou5f3.2 promote maintenance of the neuro-progenitor state rather than neurodifferentiation. Cooperativity with SoxB1-class proteins leads to inhibition of epidermis formation but expanded neural tube formation [14,78]. Co-injection of Pou5f3.1 and SoxB1 into blastomeres leads to the appearance of neuron-filled protrusions at the tailbud stage [14]. Interactions of POUV and SoxB1 in the maintenance of neural progenitors is thus reminiscent of interactions between Oct4 and Sox2 in mammals in pluripotency control. Inhibition of ectodermal formation is thought to occur through BMP suppression by Pou5f3.2 [79]. *Xenopus* Pou5f3 proteins have been found to inhibit posterior neural fate. FGF-induced Sall4 suppresses their activity and thus promotes spinal cord formation, as *Sall4* knockdown leads to Pou5f3 upregulation and loss of spinal cord tissue [80]. Of note, this is not true for overall posterior extension, as other studies indicate that *Pou5f3* depletion also leads to posterior body truncation [11,76]. Moreover, this phenotype is characteristic of both zebrafish and mammals (discussed below).

There is also a documented function of Pou5f3 proteins in inhibition of mesendoderm formation [27,78]. These proteins act in opposition to both activin/nodal and FGF signalling through inhibition of VegT/beta-catenin, Gsc and Mix2 [12,81,82]. Of note, Xbra, a marker of mesendoderm progenitors, is differentially dependent on *Pou5f3* knockdown: it is downregulated upon knockdown of all Pou5f3 proteins (PVD2 in the article) [13] while upregulated during gastrulation upon *Pou5f3.1* knockdown [12,13]. It was also shown that Pou5f3s could act as a repressor of Nodal/TGF-beta signalling by direct DNA binding of Foxh1 targets. Pou5f3 binding motifs were found in Foxh1 ChIP-seq data, and *Pou5f3* knockdown led to upregulation of *Gsc* and *Nodal2*, both controlled by Foxh1 [83]. Interestingly, mouse Oct4 behaves like its *Xenopus* orthologues, as its overexpression also leads to inhibition of mesendoderm differentiation in *Xenopus* [78]. Also, *Xenopus* Pou5f3.1 can rescue *Oct4*-deficient mouse ESC self-renewal [11] and mouse Oct4 can substitute for Pou5f3 in zebrafish development [84]. Therefore, one could conclude that POUV proteins have not undergone any principal structural changes throughout vertebrate evolution but acquired their functions according to the species-specific developmental context. Due to the differential regulatory environment, POUV protein functions may have different effects. For example, in zebrafish, POUV induces endoderm formation and in mammals, it induces primitive endoderm formation, whereas in *Xenopus*, POUV suppresses mesendoderm formation (figure 2). At the same time, the abovementioned functions in inhibition of mesendoderm formation and posterior neural tissue formation confirm a role for POUV in prevention of premature differentiation.

In addition to the described functions of Pou5f3 proteins during neuroectoderm and mesendoderm specification, these proteins play an important function in embryo integrity [13]. Livigni *et al.* compared conserved POUV targets in *Xenopus*, mouse and human, and revealed that evolutionarily conserved genomic targets are related to cell adhesion. Improved knockdown of all Pou5f3 mRNAs in this work led to complete embryo disaggregation at the neurula stage, while injection of Pou5f3.1, Pou5f3.2, or mouse Oct4 mRNA rescued this phenotype. Those authors considered the following: (1) conserved POUV genome targets are associated with cell adhesion; (2) E-cadherin overexpression partially rescues *Pou5f3* (PVD2) knockdown in *Xenopus* and blocks differentiation of mouse ESCs in the absence of the Oct4; and (3) Pou5f3.1 and Pou5f3.2 could rescue the self-renewal of mESCs at least partially by maintaining E-cadherin expression. The authors thus speculated that an ancient role of POUV-class proteins is to block delamination itself and to support an undifferentiated state via ‘uncommitted ectodermal epithelium’ [13].

## 4. Biological functions of the POUV proteins in amniotes

Amniotes are characterized by the presence of amnion during embryogenesis. Amnion is a liquid-filled structure that allows the embryo to develop in an out-of-water environment. These taxa include reptiles, birds and mammals. Unfortunately, there are just a few pieces of information about POUV proteins in non-mammalian amniotes [85]. It is known that (1) both *Pou5f1* and *Pou5f3* are present in turtles, (2) only *Pou5f1* is present in snakes and lizards, and (3) only *Pou5f3* is present in crocodiles and birds (figure 1). Pou5f1 expression was observed in the posterior segment of the snake embryo, suggesting that Pou5f1 participates in trunk elongation during snake development [86]. Early mammals such as monotremes and marsupials have both *Pou5f1* and *Pou5f3*. Other mammals harbour *Pou5f1* (*Oct4*) while some (rodents and primates) also have the relatively newly emerged *Pou5f2*, which is expressed in male germ cells [7,22,87]. Chicken *Pou5f3*, like *Nanog*, is expressed at a high level in chicken ESCs (cESCs), and, like mouse *Oct4*, its expression is downregulated upon retinoic acid treatment [88]. During early chicken development, Pou5f3 was found first in the epiblast and, to a limited extent, in hypoblast in the pre-streak embryo stage; then in the primitive streak, ectoderm, and mesoderm during gastrulation; and finally, in the neural tube, underlying the mesoderm, and in PGCs, but not in endoderm [89]. This expression pattern of *Pou5f3* is more reminiscent of its orthologues in zebrafish and *Xenopus* rather than *Pou5f1* expression in mammals, as one would expect.

Unlike the case for zebrafish and *Xenopus*, POUV research in mammals is performed by using not only an animal genetics approach but also cultured pluripotent stem cells or cellular reprogramming into a pluripotent state. Despite the longstanding comprehensive research on these proteins in mouse and human, new data continues to emerge and change our view of POUV functions in these species. While most studies underlie the function of Oct4 in mammals as a gatekeeper of the undifferentiated pluripotent state, data on Oct4 function in early differentiation begins to accumulate.

In mammalian ontogenesis, Oct4 is first detected in oocytes, and after fertilization, its transcription begins before the 8-blastomere stage; after trophectoderm segregation, Oct4 is detected in the inner cell mass (ICM) at embryonic day 3.5 (E3.5). Oct4 becomes transiently upregulated in the primitive endoderm (PrE) and is expressed in the pluripotent epiblast before (E4.5) and after implantation (E5.5-E8.0). During gastrulation and with the onset of somitogenesis, *Oct4* expression is downregulated and subsequently becomes restricted to PGCs (figure 2) [2,90–92]. Oct4 is downregulated during spermatogenesis, but spermatogonial stem cells remain positive for Oct4. Oct4 is not detected at the early stages of oogenesis but is re-expressed during the growth phase of primary oocytes and is present until fertilization [93–95]. Oct4 is a key marker of cultured pluripotent stem cells, with two major stem cell types identified: the classic so-called ‘naive’ cells, which are ESCs that correspond to and could be obtained from the epiblast before implantation [15,16,96,97]; and the ‘primed’ epiblast stem cells (EpiSCs), which correspond to the epiblast after implantation [98,99]. In the past few years, an intermediate ‘formative’ pluripotent stem cell type, which corresponds to E5.5 epiblast and to cultured epiblast-like stem cells (EpiLCs), was identified and shown to have an ability to differentiate into germ cells [100–103]. Loss of pluripotency correlates with *Oct4* downregulation in epiblast and thus, EpiSCs could be obtained up to E8.0 [92]. ESCs and EpiLCs/EpiSCs differ from each other by the presence of specific markers, signalling and culture conditions [102,104]. While ESCs are mainly dependent on leukaemia inhibitory factor (LIF), EpiLCs/EpiSCs require bFGF/Activin for self-renewal [98,99,105]. The *Pou5f1* gene is subject to complex transcriptional regulation: it has three key regulatory elements—the distal enhancer (approx. 2 kb 5′ from TSS), the proximal enhancer (approx. 1 kb 5′ from TSS), and the proximal promoter—targets of a variety of transcriptional regulators [2,90,106–108]. The distal enhancer is active in the pluripotent epiblast of preimplantation embryos and, as expected, in its cultured counterparts, ESCs, while the proximal enhancer is active in the epiblast of post-implantation embryos and the cells derived thereof, EpiSCs [109]. An additional enhancer element, which is located within the first intron of POU5F1 gene, was found to be active in naive human ESCs. This enhancer is conserved across placental animals but it is not active in mouse ESCs [110].

### 4.1. Oct4 functions in mammals before implantation

Early functional studies pointed to an Oct4 role as an antagonist to Cdx2 during morula separation into ICM (Oct4+) and trophectoderm (TE, Cdx2+) [4,17,111]. Nichols *et al.* showed that while Oct4-null blastocysts were initially established, they gave rise to only trophoblast giant cells in outgrowth experiments and no implanted mutant embryos were found at E5.5 [17]. A further study by Niwa *et al.* with a regulatable Oct4 transgene system showed that less than two-fold downregulation of Oct4 in ESCs led to ESC differentiation into trophoblast, while comparable Oct4 upregulation drove ESC differentiation into primitive endoderm (PrE) and mesoderm [4]. This study pointed to Oct4 roles in differentiation to both extraembryonic cell types—TE and PrE—via unknown mechanisms. Another work with Oct4 knockdown in ESCs showed upregulation of Cdx2, Hand1, Eomes and Mash2 mRNAs [112]. Further research revealed an important role for Cdx2 in trophoblast stem cell specification and maintenance, while Oct4 downregulation induced trophoblast differentiation normally in Cdx2-null cells [111,113]. However, more recent *in vitro* and *in vivo* studies brought to light an alternative view of the role of Oct4 levels in lineage choice. It was clearly shown that in ESCs, Oct4 level could be reduced two-fold [114], and even seven-fold, resulting in the emergence of a robust naive pluripotent state [5]. Those cells with constitutively low Oct4 level could be maintained in both defined N2B27 and serum-containing media without LIF and additional inhibitors. The authors also showed that Oct4 overexpression resulted in differentiation of ESCs into all three embryonic lineages [5]. Nonetheless, elimination of Oct4 in ESCs led to loss of pluripotency—first, ‘naive’ markers were downregulated, then, trophectodermal genes were upregulated [115]. Interestingly, at early timepoints after rapid Oct4 depletion via an auxin-inducible degron approach, Nanog binding to its genomic targets was enhanced, within both Oct4-occupied and Oct4-free regions, ruling out the possibility of physical competition between Oct4 and Nanog [115]. The derivation of maternal and zygotic Oct4-knockout mouse embryos surprisingly revealed that TE-ICM segregation and epiblast specification proceeded normally without Oct4; however, PrE formation was abolished [116–118]. These studies showed several interesting facts. First, the formation of Nanog-positive pluripotent epiblast is not affected, and at E3.0-E4.0 stage, the average number of outside and inside cells is similar between wild-type and Oct4-null embryos. Second, Cdx2 mRNA level is elevated as early as E4.5 in *Oct4*-knockout embryos, pointing to the notion that reciprocal interaction between Oct4 and Cdx2 is needed for the maintenance rather than the establishment of the ICM and TE. Third, initial PrE marker Gata6 expression is not affected; however, further Sox17 and Gata4 activation with subsequent PrE maturation is not observed in *Oct4* mutants. Finally, a recent study by Stirparo *et al.* showed that in Oct4-deficient ICM of an early blastocyst (E3.5), the TE markers Gata2, Gata3, Eomes, but not Cdx2, are upregulated [119]. Moreover, the authors pointed to failure of activation of both epiblast- and PrE-specific genes in late blastocyst (E4.5), decline in P-STAT3 level and glycolytic gene activity, and upregulation of genes associated with autophagy and lysosomes, which is most likely a response of energy-insufficient metabolism [119]. Of note, in line with the results of Livigni *et al.* on the conserved role of POUV proteins in cell adhesion [13], an enrichment in modulated genes associated with cell adhesion and tight junction formation was observed in mutant E4.5 blastocysts [119]. Considering that Pou5f3 is important for zebrafish endoderm development but is indispensable for Oct4-deficient mESC maintenance, it would be interesting to investigate whether zebrafish Pou5f3 could rescue PrE maturation in Oct4-deficient mouse embryos.

Initial work by Niwa *et al.* [4] showed another interesting feature: artificial expression of Oct4 to wild-type level in ESCs, together with LIF withdrawal, led to differentiation of ESCs into PrE, as did Oct4 overexpression in the presence of LIF [4]. LIF is a member of the IL-6 family of cytokines. Its binding to its target receptor promotes STAT3 phosphorylation, which in turn regulates Oct4 expression through the occupation of the *Pou5f1* distal enhancer [105,120]. The data by Niwa *et al*. suggest that LIF/p-STAT3 are needed to not only activate Oct4 transcription but also provide a regulatory context supportive of Oct4's role as a pluripotency gatekeeper rather than a lineage specifier. Oct4 dimerizes with Sox2, but Oct4 can also dimerize with Sox17 on the so-called compressed motifs near PrE genes [121,122]. Oct4 dephosphorylation at T343 amino acid leads to a shift in Oct4 dimerization preference, from Oct4-Sox2 dimers to Oct4-Sox17 dimers [123]. Thus, one can hypothesize that, directly or indirectly, LIF/p-STAT3 signalling modulates Oct4 activity, for example, by phosphorylation to favour Oct4 interaction with Sox2 instead of Sox17. The model can explain, for example, the robust pluripotent state of ESCs with low Oct4 level [5,114]—the decrease of the total amount of Oct4 protein may shift the equilibrium toward the phosphorylated form. However, this idea is not supported by the observation that ESCs can be maintained with seven-fold downregulated Oct4 level, even without LIF [5]. On the other hand, the ‘LIF/p-STAT3 context’ hypothesis agrees with another recent study from the same research group [124]. Following the generation of mouse chimeras by introducing ESCs with constitutive Oct4 expression, the authors could successfully obtain Oct4-expressing MEFs. These cells could be reprogrammed into iPSCs simply by addition of LIF and simultaneous transfection with IL6 and IL6 receptor-encoding constructs—i.e. by LIF/p-STAT3 pathway activation [124]. Interestingly, while *STAT3*-knockout embryos at E3.5 demonstrate normal morphology, as well as normal Cdx2, Oct4, and Nanog expression, those at E4.5 consist almost entirely of Cdx2-positive (but Oct4- and Nanog-negative cells) and very few Gata6-positive cells [120]. Of note, it appears that p-STAT3 and Oct4 regulation is consistent with the previously discussed work showing that p-STAT3 level declines in response to *Oct4* knockdown in the mouse blastocyst [119]. The role of Oct4 in PrE development is also related to Fgf4 activation. Oct4 plus Sox2 together occupy the *Fgf4* enhancer and drive *Fgf4* transcription [125]. Secreted by pluripotent epiblast, Fgf4 binds to the Fgf receptors Fgfr1 and Fgfr2, thereby activating the MAPK/ERK-signalling pathway and driving the PrE differentiation programme [126,127]. Inhibition of the FGF pathway leads to the conversion of all ICM cells into Nanog-positive epiblast cells at E4.5 [128], while addition of exogenous Fgf4 induces the conversion of ICM cells into Gata6-positive PrE cells [129].

Though the role of the Oct4 in pre-implantation development is well studied in mouse, some differences in Oct4 function are seen across other placental animals. During bovine and human Oct4-null blastocyst development, ICM-TE segregation was found to occur as in mouse but without Nanog activation [130]. It was also shown that Oct4 knockout leads to some difficulties in expansion of human blastocyst: 47% (8 of 17) of control Cas9-injected embryos developed to the blastocyst stage, whereas 19% (7 of 37) of Oct4-targeted-Cas9 embryos matured to this state [131]. There are also significant differences in the role of FGF during epiblast–PrE segregation. While activation or inhibition of the FGF pathway in mouse is critical for PrE or epiblast establishment, respectively, it has little role in bovine and no role in human pre-implantation development [132,133].

### 4.2. Oct4 functions in mammals after implantation

There is also substantial evidence about the importance of Oct4 in cell differentiation within the embryo proper. Based on zebrafish and *Xenopus* Pou5f3 studies and a one report showing that under serum-free conditions, Oct4 upregulation leads to ESC differentiation into neuroectoderm, it is possible that Oct4 plays a similar role in the neuroectoderm development of mammals [134]. However, most studies point to a key role of Oct4 in mesendoderm specification. Upregulation of Oct4 via transgenesis or by TGF-beta induction triggered differentiation toward the cardiac lineage [135]. While Sox2 and Oct4 cooperate to maintain the pluripotent state in ESCs, they act oppositely during ESC differentiation into neuroectoderm (NE) and mesendoderm (ME), respectively. At initial steps of specification of both mouse and human ESCs, Sox2 and Oct4 levels are strongly predictive of subsequent ME and NE fate—i.e. before the expression of corresponding markers [136,137]. During differentiation of mouse ESCs, Oct4 suppresses the NE state and does not colocalize with Sox1, while Sox2 antagonizes ME (Brachyury+) specification. It was also shown that ME specification is driven by Nac1 in cooperation with Oct4 and that NE specification is driven by Tcf3 and Sox2, as Nac1 and Tcf3 downregulation compromised the corresponding differentiation [138]. In human ESCs, robust Oct4 expression, in cooperation with BMP signalling, led to ME specification, while Oct4 knockdown, along with BMP repression, promoted the NE state [139]. Using mouse primed EpiSCs, Yu *et al.* demonstrated that inhibition of FGF signalling leads to Oct4 downregulation, abrogation of ME markers, and subsequent NE differentiation by a default mechanism that does not require additional factors in the media [140].

These results, along with the observation that the ME factor Gata3 can substitute for Oct4 in Yamanaka's cocktail for fibroblast reprogramming toward iPSCs, suggest that pluripotency may be maintained by antagonism of NE and ME specifiers [141,142]. Shu *et al.* proposed a ‘seesaw’ model that postulates that a delicate balance between Oct4 and Sox2 levels prevents differentiation into NE or ME [141]. However, this hypothesis was refuted by several facts, as discussed in a recent work by Velychko *et al.* [143]. First, forced expression of Oct4 could rescue the pluripotent state in Sox2-knockout ESCs [144]. Second, Velychko and colleagues showed that only KSM—that is, the reprogramming cocktail without Oct4—could reprogram fibroblasts into iPSCs, and that Oct4 and Gata3 work primarily to enhance proliferation during reprogramming [143]. Finally, to the best of our knowledge, there were no successful attempts to obtain stable ESC lines with substitution of Oct4 and Sox2 by ME and NE factors, for example, by Brachyury and Sox1, respectively.

An interesting difference is observed in Oct4 partner choice during PGC maturation in mouse and human. Sox2 is expressed throughout the establishment of murine PGCs [100], whereas Sox17 substitute for Sox2 in human PGCs [145]. As it was pointed above, the Sox17-Oct4 complex occupies compressed motifs, which are 1 bp shorter that the canonical Sox2-Oct4 motif [121]. Analyses of open chromatin during hPGC maturation have revealed the compressed Sox-Oct motif occurs across active DNA elements [146,147]. As Sox17 is associated with endoderm specification, the rationale for Oct4 partner switching from Sox2 to Sox17 in human PGC development remains unclear. This may have to do with differences in the mechanism of germ cell differentiation between mouse and human. While mouse germ cells are induced from the murine primed EpiLCs, human ESCs are supposed to go through the so-called pre-mesendoderm condition [148].

Although *in vitro* works usually point to rather definite roles for Oct4 in the specification of one or another cell type of the embryo proper, *in vivo* data are more comprehensive. Depletion of Oct4 in PGCs by Cre/loxP gene targeting led to apoptosis of these cells between E9.5 and E10.5, pointing to a specific Oct4 role in PGC viability and maturation [94]. Further development of germ cells differentially requires Oct4 expression. Spermatogonial stem cells depend on Oct4 for their self-renewal, and Oct4 knockdown leads to a reduced ability to colonize the seminiferous tubules [149]. ZP3-dependent Oct4 depletion in growing oocytes do not affect oocyte maturation and embryogenesis immediately after fertilization (discussed above) [116]. In other works, DeVeale *et al.* and Mulas *et al.* used conditional systems that allowed them to knock out Oct4 after embryo implantation and thus, to dissect Oct4 function in the early gastrulating embryo [19,150]. Oct4 depletion from approximately E7.0 onward led to multiple defects— craniorachischisis, posterior truncation, random heart tube orientation, defective somitogenesis and failed anterior–posterior orientation. Mulas *et al.* demonstrated that in Oct4-null epiblasts, Nanog expression was elevated and endoderm was expanded at the expense of mesoderm [150]. An interesting observation of this work is that beating structures were successfully obtained from Oct4-depleted embryos, pointing to a successfully launched mesendoderm programme and, again, contradicting the view that Oct4 is a factor indispensable for differentiation into ME. However, the epithelial-to-mesenchymal transition was impaired due to E-cadherin upregulation and overall, the anterior-posterior axis was abnormal. DeVeale *et al.* also pointed to an Oct4 role in primitive streak (mesendoderm) proliferation and suggested a conserved Oct4 function in posterior extension, consistent with the observed posterior truncations in both zebrafish and *Xenopus* POUV mutants [11,18,65]. The data is also in accordance with the demonstrated Oct4 role in regulation of the trunk length in snakes and mice. While prolonged Oct4 expression in mice (via Cdx2 enhancer-driven posterior expression up to E12.5) led to an abnormal increase in number of ribs, exceptionally long snake trunks might be a result of heterochronic changes in Oct4 activity during body axis extension [86].

Finally, a very recent study revealed that Oct4 is reactivated in premigratory cranial neural crest cells (CNCCs) at early somitogenesis (E8.0), endowing CNCCs with the pluripotent state [151]. CNCCs present a specific population of cells with ectodermal origin and are responsible for craniofacial skeleton development—not only neurons and glia but also bone, cartilage, and muscles. In their study, Zalc *et al.* demonstrated that Oct4 ablation at E7.5 leads to complete absence of the front nasal mass. The pluripotent state was confirmed by the observation that in the absence of Oct4, neural derivatives of CNCCs developed normally, while ectomesenchyme maturation was affected. Furthermore, ATAC-seq of Oct4-positive CNCCs revealed that these cells clustered with EpiSCs, while the Oct4-positive trunk cells, which were also present during early somitogenesis, did not [151]. Notably, artificially maintained constitutive Oct4 levels do not prevent any type of differentiation during chimera formation up to E12.5 of mouse development [5,124]. Overall, as we discussed earlier with an example of LIF/p-STAT3 signalling, it appears that Oct4 is a context-dependent transcription factor. In some cases, it safeguards pluripotency, while in other cases, it maintains proliferation or at least does not prevent differentiation.

There were numerous reports about Oct4 expression in different somatic cells of the adult organism, prompting Lengner *et al.* [152], using a Cre/lox-based genetic approach, to examine the role of Oct4. The authors found that Oct4 does not play a role in the compartments of several somatic tissues, such as intestinal epithelium, bone marrow (haematopoietic and mesenchymal lineages), hair follicle, brain and liver [152]. The investigation appeared to settle the debate about whether Oct4 functions outside of the mammalian germline (epiblast and PGCs); however, a recent study again sparked the debate. In that study, investigators relied on a similar Cre/lox-based genetic approach and the same *Oct4^flox^* mouse line and demonstrated that Oct4 is induced in mouse atherosclerotic lesions. Oct4 expression was observed in smooth muscle cells (SMCs) and thought to promote a specific atheroprotective SMC phenotype switch involved in the formation of a protective fibrous cap. SMC-specific depletion of Oct4 led to an increase in the size of atherosclerotic lesions and, consequently, in reduced lumen size, increased necrotic core area, and increased intraplaque haemorrhage [153]. It might be that under specific pathological conditions, adult somatic cells can engage Oct4 function, and the reported case with atherosclerotic SMCs might not be unique in that respect.

## 5. Conclusion

Despite the huge amount of data about POUV-class proteins, there are still a lot of questions about their origin and mechanisms of their action. Absence of any POUV member in lampreys complicates our understanding about the emergence of this class. Considering the similar early development of lamprey and zebrafish, and the early lethal phenotype of zebrafish with *Pou5f3* knockout, it is unclear how lampreys develop without POUV. Future works should look to uncover whether there are some alternative regulatory mechanisms that enable normal development in the absence of POUV or that other proteins present in these organisms control the same processes.

It appears that the main function of the POUV-class proteins is maintenance of the undifferentiated state through activation of their genome targets. Though there was some evidence about their role in transcriptional repression, it was also shown that specific protein fusion making only the active form of *Xenopus* Pou5f3 or mouse Oct4 is sufficient for performing the whole range of functions [154]. The involvement of POUV proteins in different processes enables maintenance of the undifferentiated state. During *Xenopus* neuroectoderm development, Pou5f3 function is related to the maintenance of neural progenitors [14] and prevention of ectodermal differentiation [79]. The conservative role of POUV in posterior extension [11,18,19] also indicates that proteins of this class prevent immature differentiation, delaying the onset of further body patterning, for example, by prevention of *Hox* genes activation [86]. Finally, as the most notable member of the POUV class, Oct4 functions in maintenance and induction of the undifferentiated state, thus being a key determinant of mammalian pluripotent stem cells [3,17,155,156].

The facts indicate that the mechanisms of POUV-class protein functioning are species and stage dependent. As we have previously discussed [8], these proteins are inextricably linked to zygotic genome activation in zebrafish and *Xenopus*, and, to a limited extent, in mammals. The ability of murine Oct4 to rescue Pou5f3-deficient zebrafish in early development [84] or *Xenopus* Pou5f3 to substitute for Oct4 in mouse ESCs [11] indicates that POUV class homologues did not undergo significant structural changes. At the same time, Pou5f3 from zebrafish could not rescue the pluripotent properties of murine ESCs [11]. Moreover, while Pou5f3 is needed for endoderm establishment in zebrafish [26,65], Pou5f3 orthologues suppress mesendoderm maturation in *Xenopus* [11,27]. Additionally, POUV could exert a different function in the same species depending on the developmental stage. A striking example of context-dependent activity is the behaviour of murine Oct4 during embryogenesis. While murine Oct4 maintains the pluripotency of the epiblast [17,119] and induces this cell state in CNCCs [151], when introduced artificially, it does not prevent any type of differentiation at subsequent development up to E12.5 [124]. Moreover, when Oct4-expressing MEFs were isolated at this stage, they were successfully reprogrammed into iPSCs only via LIF/p-STAT3 pathway activation. It appears that to arrive at a better understanding of the POUV-class protein function, one should take into consideration their functional amino acids and the regulatory environment engaged depending on the developmental stage.

**Figure 3.**
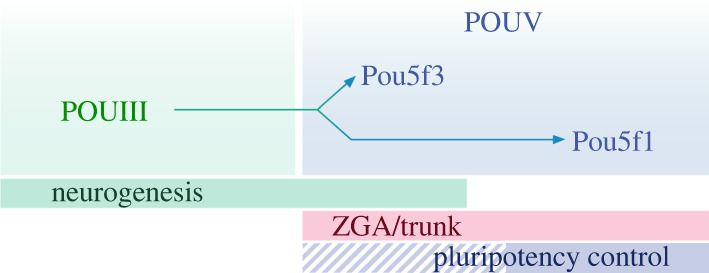
Functional diversity between POUIII-class ancestor and POUV-class orthologues—Pou5f1 and Pou5f3.

We have also previously discussed that the functional novelty of the POUV class is the ability to dimerize *in vivo* with proteins of the SoxB class. This property was immediately linked to participation of POUV members in ZGA (figure 3) [75,76]. It is most likely that POUV-class proteins have emerged from some of the POUIII-class proteins, which are known to be regulators of neuroectodermal development. Taking this into account, it appears that the function of zebrafish and *Xenopus* Pou5f3 in neurogenesis is an evolutionary inherited feature. The Pou5f1 orthologue (Oct4), which is present in mammals, has probably lost this feature, as it acts primarily in pluripotency control [5] and mesendoderm development [137,150]. Nevertheless, murine ESCs, which harbour only this Pou5f1 orthologue, are characterized by epithelial morphology, E-cadherin expression, and the default capacity for neuroectodermal differentiation [157]. These features, along with the ancient role of POUV-class proteins in preventing differentiation by securing uncommitted ectodermal epithelium [13], point to a close link between ectoderm and cells expressing POUV, enhancing our understanding of the nature of cellular pluripotency.

## Data Availability

This article has no additional data.

## Abbreviations

POU: name of transcription factor class, which name is originated by first letters of the names of the founding members of the family—mammalian Pit1, Oct1, Oct2, and *C. elegans* Unc86
ZGA: zygotic genome activation
POUs: POU-specific subdomain
POUh: POU-homeodomain
WGD: whole-genome duplication
PGCs: primordial germ cells
MHB: midbrain hindbrain boundary
MZspg: maternally and zygotically knocked-out Pou5f3 (abbreviation for studies with zebrafish)
PVD2: ‘POUV-depleted 2’, designation of the morpholino antisense oligos combination for POUV-proteins knockdown in *Xenopus* [13]
ICM: inner cell mass
PrE: primitive endoderm
TE: trophectodem
ESCs: embryonic stem cells
EpiSCs: epiblast stem cells
EpiLCs: epiblast-like stem cells
LIF: leukaemia inhibitory factor
NE: neuroectoderm
ME: mesendoderm
CNCCs: cranial neural crest cells
