# Correction to: ‘The functional diversity of the POUV-class proteins across vertebrates’ (2022) by Bakhmet and Tomilin

**DOI:** 10.1098/rsob.220306

**Published:** 2022-11-09

**Authors:** Evgeny I. Bakhmet, Alexey N. Tomilin

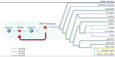

*Open Biology* **12**, 220065. (Published online 29 June 2022) (https://doi.org/10.1098/rsob.220065)

The fragments of the review listed below are incorrect due to novel information about POUV-gene presence in cyclostomes (lampreys and hagfish):

**Section 1. Introduction:** ‘Surprisingly, though, members of the POUV class were found only in vertebrates and, remarkably, only in jawed vertebrates (Gnathostomata), from cartilaginous fishes to human, and not in lampreys, for example [6,7].’

**Section 2. POUV-class origin:** ‘Two subsequent WGDs took place between tunicates and lampreys [33,34] and perhaps account for the switching from a “mosaic” type of development to a “regulative” development. The former type of development is typical for most invertebrates while the latter applies to all vertebrates. However, the absence of any POUVs in the lamprey's genome [35] does not support the role of WGDs in the origin of the POUV class. Thus, unless lampreys had POUV genes and then lost them, this class likely appeared by simple duplication of some POUIII gene (figure 1) [36]. This is confusing in light of the relatively similar early development of lamprey and zebrafish [37,38] as well as the indispensability of POUV-class proteins for embryogenesis of all vertebrates except lampreys (discussed below).’

**Figure 1.**
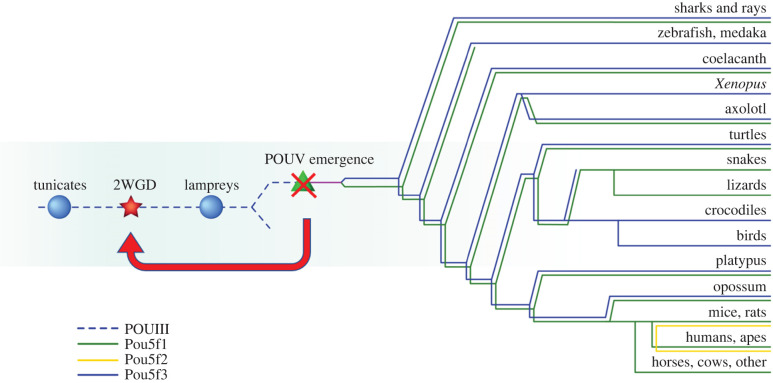

**Section 5. Conclusion:** ‘Absence of any POUV member in lampreys complicates our understanding about the emergence of this class. Considering the similar early development of lamprey and zebrafish, and the early lethal phenotype of zebrafish with Pou5f3 knockout, it is unclear how lampreys develop without POUV’.

At the moment of the manuscript preparation, we had no information about POUV proteins in cyclostomes. Our attempted search for POUV members also was unsuccessful. However, a recent study by Woranop Sukparangsi and colleagues clearly demonstrates that cyclostomes including lampreys bear at least one gene from this class [1].

So far, there are no inconsistencies with the absence of POUV proteins in lampreys. It seems that these proteins could indeed emerge during whole-genome duplications and could make a contribution to switching from a 'mosaic' type of development to a 'regulative' development.

Though Sukparangsi *et al*. point that POUV proteins from cyclostomes do not support the pluripotency, in our view, these proteins still could contribute to zygotic genome activation (ZGA). The role of POUV in ZGA is discussed in our previous review [2].
